# From Epidemiology of Community-Onset Bloodstream Infections to the Development of Empirical Antimicrobial Treatment-Decision Algorithm in a Region with High Burden of Antimicrobial Resistance

**DOI:** 10.3390/antibiotics12121699

**Published:** 2023-12-05

**Authors:** Darunee Chotiprasitsakul, Akeatit Trirattanapikul, Warunyu Namsiripongpun, Narong Chaihongsa, Pitak Santanirand

**Affiliations:** 1 Division of Infectious Diseases, Department of Medicine, Faculty of Medicine Ramathibodi Hospital, Mahidol University, Bangkok 10400, Thailand; akeatit.tri@mahidol.ac.th (A.T.); warunyu.nam@mahidol.ac.th (W.N.); 2 Microbiology Laboratory, Department of Pathology, Faculty of Medicine Ramathibodi Hospital, Mahidol University, Bangkok 10400, Thailand; narong.chh@mahidol.ac.th (N.C.); pitak.san@mahidol.ac.th (P.S.)

**Keywords:** bacteremia, antimicrobial resistance, epidemiology, community, Asia

## Abstract

Antimicrobial-resistant (AMR) infections have increased in community settings. Our objectives were to study the epidemiology of community-onset bloodstream infections (BSIs), identify risk factors for AMR-BSI and mortality-related factors, and develop the empirical antimicrobial treatment-decision algorithm. All adult, positive blood cultures at the emergency room and outpatient clinics were evaluated from 08/2021 to 04/2022. AMR was defined as the resistance of organisms to an antimicrobial to which they were previously sensitive. A total of 1151 positive blood cultures were identified. There were 450 initial episodes of bacterial BSI, and 114 BSIs (25%) were AMR-BSI. Non-susceptibility to ceftriaxone was detected in 40.9% of 195 *E. coli* isolates and 16.4% among 67 *K. pneumoniae* isolates. A treatment-decision algorithm was developed using the independent risk factors for AMR-BSI: presence of multidrug-resistant organisms (MDROs) within 90 days (aOR 3.63), prior antimicrobial exposure within 90 days (aOR 1.94), and urinary source (aOR 1.79). The positive and negative predictive values were 53.3% and 83.2%, respectively. The C-statistic was 0.73. Factors significantly associated with 30-day all-cause mortality were Pitt bacteremia score (aHR 1.39), solid malignancy (aHR 2.61), and urinary source (aHR 0.30). In conclusion, one-fourth of community-onset BSI were antimicrobial-resistant, and one-third of *Enterobacteriaceae* were non-susceptible to ceftriaxone. Treatment-decision algorithms may reduce overly broad antimicrobial treatment.

## 1. Introduction

Bloodstream infections (BSIs) are a leading cause of morbidity and mortality globally [1,2]. The epidemiology of BSI has evolved and differs considerably between developed and developing countries. Asia is considered a high burden region of antimicrobial resistance [3]. Multidrug resistance was presented in 30% of cases of Gram-negative bacteremia in a community hospital in Thailand [4]. The mortality rate of BSI varies from 12% for community-onset BSI [5], 22% in a population-based cohort study [6], 30% for patients who have severe comorbidities, and 40–60% for intensive care unit patients [2]. Increasing antimicrobial resistance (AMR) is a significant cause of death worldwide. The highest burden is in low-resource settings [7]. Mortality in *Escherichia coli* and *Klebsiella* BSI strongly depends on resistance to fluoroquinolones or third-generation cephalosporins and on adequate therapy [8]. The median turnaround times were 0.80, 1.81, and 2.71 days for Gram stain, identification of organism, and antimicrobial susceptibility test (AST) results, respectively [9]. A prompt selection of empirical antimicrobial treatment without knowing whether an infection is antimicrobial-resistant, while balancing the risk of ineffective treatment versus excessively broad antimicrobial therapy is difficult. Understanding the extent of community-onset BSIs would help address the magnitude and impact of AMR and develop a solution.

We aimed to describe the contemporary epidemiology and outcomes of bacteremia in a community setting at emergency room (ER) and outpatient clinics. Our secondary objective was to identify independent factors associated with AMR in patients with BSI and develop the empirical antimicrobial treatment-decision algorithm for patients with suspected community-onset bloodstream infections.

## 2. Results

### 2.1. Distribution of Pathogenic Organisms Associated with Community-Onset Bloodstream and Antimicrobial Susceptibility

**Figure 1:**
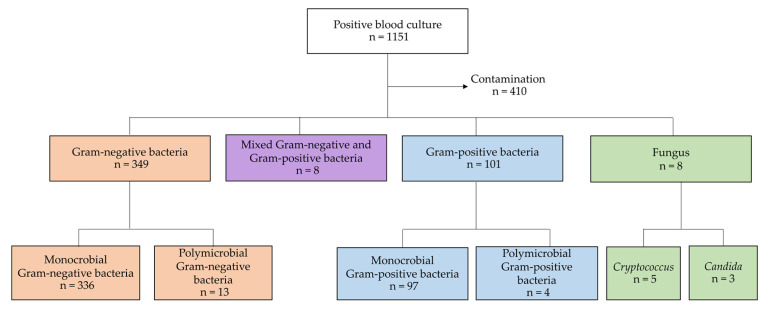
Distribution of pathogenic organisms associated with community-onset bloodstream infection.

A total of 1151 positive blood cultures were identified. Of these, 410 specimens were contaminants. There were 460 initial episodes of BSI; 336 monomicrobial Gram-negative, 13 polymicrobial Gram-negative, 97 monomicrobial Gram-positive, 4 polymicrobial Gram-positive, 8 mixed Gram-negative and Gram-positive, 5 *cryptococcus*, and 3 *candida* (Figure 1). Of 487 unduplicated bacterial isolates with AST, the most common organisms were *Escherichia coli* (40.0%), followed by *Klebsiella pneumoniae* (13.8%), *Staphylococcus aureus* (7.6%), beta-hemolytic streptococcus (5.3%), *Salmonella* spp. (3.7%), and *Pseudomonas aeruginosa* (3.7%). Non-susceptibility to ceftriaxone was identified in 40.9% of 195 *E. coli* isolates and 16.4% among 67 *K. pneumoniae* isolates. All 18 *P. aeruginosa* isolates were susceptible to ceftazidime. Among the Gram-positive bacteria, 5.4% of 37 *S. aureus* isolates were methicillin-resistant, and 17.6% of 17 viridans streptococci isolates were non-susceptible to penicillin and ampicillin. Pathogens with ≥5 first isolate counts and antibiograms are summarized in Figure S1 in the Supplementary Data.

### 2.2. Clinical Characteristics of Patients with Antimicrobial-Resistant BSIs (AMR-BSIs) and Non-Antimicrobial-Resistant BSIs (NAMR-BSIs)

A cohort of 450 unique adult patients with bacterial BSIs with AST were identified during the study period and included in the comparative analysis for risk factors associated with AMR. Of these, 114 BSIs (25%) were antimicrobial-resistant. The baseline characteristics of AMR-BSI compared to NAMR-BSI patients are shown in Table 1. The majority of bacterial BSIs were detected at ER (70%). The median age in the AMR-BSI group and NAMR-BSI group was 74 years (interquartile range [IQR] 57–83 years) and 71 years (IQR 59–80 years), respectively. The severity of the acute illness index was not different between the two groups. Both groups had a Pitt bacteremia score of 1 (IQR 0-2). Some differences between the two groups were observed. Patients with AMR-BSI were more likely to have a neurological disease, connective tissue disease prior admission, colonization or infection with multidrug-resistant organisms (MDROs), previous antimicrobial exposure within 90 days, and a higher proportion of urinary sources.

### 2.3. Analysis of Risk Factors Associated with AMR and 30-Day All-Cause Mortality in Patients with Community-Onset Bloodstream Infections

In the multivariate analysis, presence of MDROs during the preceding 90 days (adjusted odds ratio [aOR] 3.62; 95% CI 1.95–6.75; *p* < 0.001), prior antimicrobial exposure within 90 days (aOR 1.94; 95% CI 1.08–3.50; *p* = 0.03), and urinary source of bacteremia (aOR 1.78; 95% CI 1.06–3.01; *p* = 0.03) were independent factors for antimicrobial-resistant infection (Table 2).

The 30-day all-cause mortality in the AMR-BSI and NAMR-BSI groups were 6.5% and 9.1%, respectively (HR 0.71; 95% CI 0.31–1.62; *p* = 0.41). Inactive empirical treatment within 24 h was not associated with 30-day all-cause mortality (HR 0.67; 95% CI 0.20–2.19; *p* = 0.51). Factors significantly associated with 30-day all-cause mortality in the multivariate model were Pitt bacteremia score (HR 0.71; 95% CI 1.20–1.62; *p* < 0.001), solid malignancies (HR 2.62; 95% CI 1.30–5.24; *p* = 0.007), and urinary source (HR 0.30; 95% CI 0.11–0.79; *p* = 0.015) (Table 3).

### 2.4. Appropriateness of Antimicrobial Use

There were 441 unique adult patients with bacterial BSI receiving at least one dose of an antimicrobial. Of 328 patients with NAMR-BSI, 190 (57.9%) received empirical treatment with broad spectrum active coverage. In total, 34 (30.1%) patients in 113 AMR-BSI patients were empirically treated with inactive spectrum coverage. Appropriate definitive treatment was not significantly different between AMR-BSI and NAMR-BSI patients. Optimal drug and duration in the AMR-BSI and NAMR-BSI groups were 33.0% and 36.3%, respectively. The median duration of treatment was 13 days in both groups. The possibility of shortening treatment duration in the AMR-BSI group and NAMR-BSI group were 57.8% and 50.2%, respectively (Table 4).

### 2.5. Proposed Empirical Antimicrobial Treatment Algorithm for Patients with Suspected Community-Onset Bloodstream Infections

**Figure 2:**
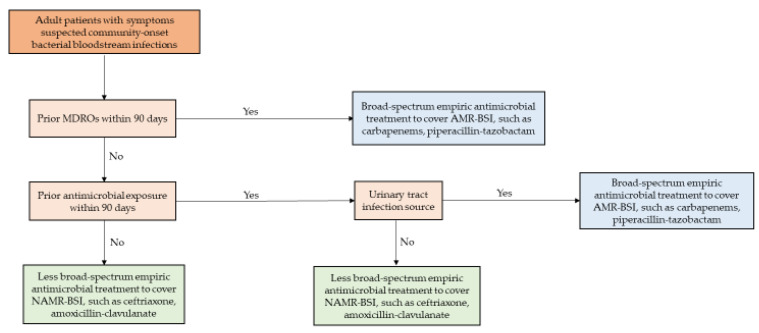
Proposed empirical antimicrobial treatment algorithm for patients with suspected community-onset bloodstream infections. Abbreviations: AMR-BSI, antimicrobial-resistant bloodstream infection; NAMR-BSI, non-antimicrobial-resistant bloodstream infection, MDROs, multidrug-resistant organisms.

Important risk factors of AMR from the analysis were integrated into treatment-decision algorithms. The following triage steps were created: identifying patients with clinical symptoms and signs suspecting bacterial BSI and stratifying patients by risk of AMR. Patients with the highest risk were defined as those who had MDROs during the preceding 90 days. These patients would be treated with broad spectrum antimicrobials. Patients not meeting this definition would be reviewed for prior antimicrobial exposure within 90 days; those with no exposure would be treated with narrower spectrum antimicrobials. Patients previously exposed to antimicrobials within 90 days would be evaluated for the suspected source of infection; those with suspected urinary source would be treated with broad- spectrum antimicrobials, and those with suspected non-urinary source would be treated with narrower spectrum antimicrobials. The development of a treatment-decision algorithm is depicted in Figure 2. The sensitivity and specificity of the algorithm for predicting AMR-BSI were 49.1% (95% CI 36.9–58.7%) and 85.4% (95% CI 81.2–89.0%), respectively. The PPV and NPV were 53.3% (95% CI 45.4–61.1%) and 83.2% (95% CI 80.4–85.6%), respectively. A receiver operating characteristic curve (ROC) curve derived from a logistic regression comprising the three most important variables yielded a C-statistic of 0.73.

Empirical treatment following the algorithm resulted in 14.9% of patients with NAMR-BSI receiving broad spectrum active coverage, and 17.7% of patients with AMR-BSI receiving inactive spectrum coverage.

Sensitivity analyses were performed on the subset of 298 patients who had *Enterobacteriaceae* BSIs. The three significant risk factors for AMR remained similar to the entire dataset. The sensitivity and specificity of the algorithm for predicting ceftriaxone-resistant BSI were 57.5% (95% CI 46.8–67.6%) and 81.9% (95% CI 75.9–86.9%), respectively. The PPV and NPV were 59.3% (95% CI 50.7–67.2%) and 80.7% (95% CI 76.6–84.2%), respectively. The C-statistic was 0.76. The internal validation of 227 bacterial BSIs revealed a C-statistic of 0.77 (Table 5). 

## 3. Discussion

The most common pathogens of community-onset BSI at our institution included *E. coli*, *K. pneumoniae*, and *S. aureus*. This is consistent with previous reports of community-acquired bacteremia in Thailand and other countries [10,11,12]. These top three pathogens accounted for 61.4% of all bacterial isolates. Other leading pathogens were beta-hemolytic streptococcus and *Salmonella* spp., which were different from previous studies [10,11,12]. A surveillance of community-acquired bacteremia in Thailand during 2016–2017 found that *E. coli* and *K. pneumoniae* had susceptibility rates to ceftriaxone of 73% and 98%, respectively [10]. In contrast, our study revealed lower susceptibility rates for these bacteria with susceptibility rates to ceftriaxone of 60.1% and 83.6%, respectively. The incidence of antimicrobial resistance continues to rise with a change driven by an increase in community-onset cases. The incidence of ceftriaxone-resistant *Enterobacteriaceae* infection increased by 53% from 2012 to 2017 according to the US Centers for Disease Control and Prevention [13]. 

In the present study, the independent risk factors for antimicrobial-resistant BSI included the presence of MDROs within 90 days, prior antimicrobial exposure within 90 days, and urinary sources in our study. These factors were similar to those identified in previous studies [3,14]. Antimicrobial selective pressure was linked to bacterial resistance [15]. High rates of community-onset antimicrobial-resistant infection have been occurring worldwide, predominantly in urinary tract infection with *E. coli* [16]. Ceftriaxone-resistant uropathogens were isolated in 21.3% of patients with acute cystitis in Thai general practice clinics from 2014 to 2016 [17]. Similarly, a study at a tertiary care hospital in Thailand reported that 22.3% of *E. coli* causing community-acquired UTI were ceftriaxone-resistant in 2017 [18]. 

The Pitt bacteremia score and solid malignancies were associated with an increase in the overall 30-day mortality in our study. The severity of illness has been well established in predicting mortality [19]. All patients with solid tumors who died during the 30-day follow-up period in our study had advanced-stage malignancy. Despite no survival advantage, antibiotics were administered in 82% of patients with terminal cancer within three days of death at an academic hospital in a retrospective study conducted in Korea [20]. Appropriately directed palliative care can reduce aggressive antimicrobial use near the end of life. It would benefit individual patients’ quality of life and decrease selection pressure that can lead to MDROs. The majority of inactive empirical antimicrobials in our study were against ceftriaxone-resistant Gram-negative BSI. Studies have shown mixed results regarding the association between the effectiveness of empirical antimicrobial treatment and ceftriaxone-resistant Gram-negative bacteremia [14,21,22]. The finding that inadequate empirical antibiotic treatment does not significantly impact the mortality in our study is consistent with previous studies [14,22]. The mortality in our study was below 10%, which is comparable to previous studies [14,22]. The patients in our study were not severely ill (median Pitt bacteremia score of 1). Relatively low mortality in community-onset bacteremia could be primarily driven by underlying conditions and disease severity [22]. The impact of empirical antimicrobial choice on mortality may be limited in this scenario. Urinary source was significantly associated with lower mortality in our study. A multi-center study in English acute hospitals found that patients with urinary tract-related bacteremia were less acutely unwell [22]. Piperacillin-tazobactam (TZP) is commonly used as an empirical treatment in our setting. The multinational, randomized, controlled trial of patients with ESBL-producing bacteremia (MERINO study) showed that definitive treatment with TZP increased 30-day mortality compared to meropenem, and no difference in mortality between urinary versus non-urinary source [23]. However, Sharara et al. reported no differences between TZP versus carbapenems in the clinical resolution or mortality for the treatment of ESBL-producing pyelonephritis [24]. A urinary pharmacokinetics study found that high TZP concentrations in urine and could result in treatment efficacy [25]. A small randomized trial showed that indwelling catheter replacement before initiating antimicrobial therapy significantly decreased bacteriuria and time to clinical improvement [26]. High TZP concentration in urine and biofilm removal in catheter-associated UTI may contribute to better outcomes compared to non-urinary-source infections. 

The most frequent inappropriate prescribing was empirical broad spectrum antimicrobial treatment was 57.9% among NAMR-BSI in our study. A study evaluating practice at ER reported that inappropriate antimicrobial prescription in adult patients was 36.9% [27]. Short courses of antimicrobial therapy (6–10 days) have been shown to have comparable clinical outcomes as prolonged courses of therapy (11–16 days) for Gram-negative bacteremia [28]. The median duration of antimicrobial treatment for the uncomplicated bacterial BSIs was 13 days in our study; shorter courses were possible in more than half of cases in our study. Although we have institutional empirical treatment guidelines and weekly handshake stewardship in the ER, there is a more pressing need to develop initiatives to improve ER-based antimicrobial prescribing and emphasis on optimal treatment duration [29]. 

Potential risk factors driving the emergence of antimicrobial-resistant bacterial infections have been identified in various studies. However, integration of multiple risk factors into actual practice is scarce. Clinicians continue to face a significant challenge when treating serious Gram-negative infections due to the difficult balance between the risk of ineffective agents versus overly broad empiric antimicrobial treatment. A prior study developed an easy-to-use clinical decision algorithm to determine the probability of an extended-spectrum beta-lactamase (ESBL)-producing bacterial BSI in a bacteremic patient that could aid in selecting appropriate empiric treatment [3]. However, it could not be applied in regions with high ESBL prevalence. 

From the analysis of risk factors for antimicrobial resistance, we developed a decision tree algorithm with three predictors; the presence of MDROs within 90 days, antibiotic exposure in the previous 90 days, and urinary tract infection source. There is always a trade-off between sensitivity and specificity. The ability to correctly predict NAMR-BSI cases (specificity) is essential to ensure the lowest risk of ineffective therapy. Patients classified as AMR-BSI cases by the algorithm were 53.3% more likely to be true AMR-BSI cases (PPV), and patients classified as NAMR-BSI cases were 83.2% more likely to be true NAMR-BSI (NPV). The subset of patients with *Enterobacteriaceae* BSIs yielded 6% higher PPV and 2.5% lower NPV. Empirical treatment following the algorithm would reduce broad spectrum antimicrobial therapy in NAMR-BSI cases by 43.0% and inactive spectrum antimicrobial in AMR-BSI cases by 12.4% in this dataset. This easy-to-use algorithm could improve the prediction of AMR-BSIs and reduce inappropriate empirical antimicrobial use. However, this algorithm cannot replace clinical judgment. Relevant components, such as clinical appearance, underlying conditions, and concern level of clinicians should be incorporated into decision-making. 

There are several limitations in our study. First, this study was conducted at a single center. Our results may not be generalizable to patients in other settings with different prevalences of antimicrobial-resistant bacteria. Our findings should be validated in other cohorts. Second, the presence of MDRO was reviewed from our clinical microbiology laboratory reports, and previous antimicrobial exposure was retrospectively reviewed from the medical records. However, cases visiting outpatient clinics and ER were usually established patients who had received healthcare services at our hospital, and missing data on these two independent factors were likely small. Finally, the broad clinical approach included both Gram-positive and Gram-negative bacteria in our algorithm. Ceftriaxone is recommended and the most commonly used empirical treatment for community-acquired sepsis at our institution. Many Gram-positive bacteria non-susceptible to penicillin are susceptible to ceftriaxone. A subset of AMR-BSI predicted by this proposed algorithm would include ceftriaxone-susceptible Gram-positives, in which ceftriaxone is reasonable to use as an empirical treatment. We performed the sensitivity analyses on *Enterobacteriaceae* BSIs, which yielded 6% increased PPV and 2.5% decreased NPV. This finding suggests that the algorithm is robust to Gram-positive and *Enterobacteriaceae*. This broad-range approach would better represent real-world practice when the initial presentation cannot distinguish between Gram-positive versus Gram-negative organisms.

## 4. Material and Methods

### 4.1. Study Population

We conducted a retrospective cohort study at Ramathibodi Hospital, a 1300-bed tertiary-care hospital in Bangkok, Thailand, between 1 August 2021 and 15 April 2022. All positive blood cultures from patients aged > 18 years at ER and outpatient clinics were identified. Only the initial episode of bacterial BSI with AST results was included in the comparative analysis of risk factors for antimicrobial-resistant infection.

### 4.2. Data Collection

Information regarding demographics, pre-existing medical conditions, and the severity of acute illness on day 1 of BSI, including Quick Sequential Organ Failure Assessment (qSOFA) score, Pitt bacteremia score, intensive care unit (ICU) admission, mechanical ventilation, vasopressor administration, receipt of antimicrobial treatment during preceding 90 days, antimicrobial-resistant bacterial colonization or infection during the prior 90 days, and microbiological and mortality data were obtained from medical records. Mortality and cause of death were assessed at 30 days. Duplicate isolates of the same species with the identical AST profile recovered from consecutive blood cultures on the same patient after the index BSI were excluded from cumulative AST.

### 4.3. Definitions

Community-onset BSI refers to the location of the onset of BSI episodes which includes community and long-term healthcare facilities. BSI is defined as the positive growth of the organism (s) from blood specimen (s) in ≥1 blood culture bottle taken from a patient with compatible clinical features of infection. The isolated bacteria are classified as contaminants if they are common commensal organisms on the skin or environment e.g., coagulase-negative staphylococci, *Bacillus* spp., *Corynebacterium* spp., *Propionibacterium* spp., *Aerococcus* spp., *Micrococcus* spp., and the patient has no compatible clinical syndrome that could be caused by such organisms. Polymicrobial BSI is defined as isolation of ≥2 different pathogens from the same blood sample. The source of BSI is determined based on clear clinical evidence that the BSI was linked to focal infection at another body site.

Antimicrobial resistance is the resistance of organisms to an antimicrobial to which they were previously sensitive. For the purpose of this study, antimicrobial-resistant bacteria are defined as follows; Gram-negative bacteria other than *P. aeruginosa* that exhibit in vitro non-susceptibility to ceftriaxone, *P. aeruginosa* that exhibits in vitro non-susceptibility to ceftazidime, and Gram-positive bacteria that exhibit in vitro non-susceptibility to penicillin-class drugs (penicillin, ampicillin, or oxacillin).

### 4.4. Microbiological Testing

All isolates were tested for their antimicrobial susceptibility by an automated microbroth dilution testing system (Sensititre™; Thermo Fisher Scientific, Cleveland, OH, USA). Clinical and Laboratory Standards Institute (CLSI) clinical breakpoints were used to interpret the minimum inhibitory concentration (MIC) values [30].

### 4.5. Assessment of the Appropriateness of Antimicrobial Use

Independent adjudication of the appropriateness of antimicrobial treatment was performed based on institutional antimicrobial guidelines by three infectious disease specialists. The local antimicrobial guidelines for common bacterial infections consisted of the recommended antimicrobials for empirical therapy and the recommended dosage of each antimicrobial [29]. Antimicrobial treatment was considered active when isolated pathogens were susceptible in vitro to the prescribed antimicrobial. Antimicrobial de-escalation or escalation encompassed the antimicrobial change within 48–72 h of available culture and susceptibility test results. 

All BSI episodes wherein at least one antimicrobial was prescribed were randomized, and each was assigned to two specialists. Each expert independently assessed the antimicrobial prescription into specific categories for appropriate antimicrobial use modified from a previous study [31] (Table S1 in the Supplementary Data).

### 4.6. Statistical Analysis

Continuous variables were summarized as the mean (standard deviation) for normally distributed data or median (interquartile range) for non-normally distributed data. Categorical variables were displayed using absolute counts and percentages. Comparative analysis of variables associated with antimicrobial resistance was conducted using the Student’s t-test or Wilcoxon rank-sum tests for continuous variables. The Chi-square test or Fisher exact test was used to compare categorical variables. Logistic regression was used to calculate the odds ratios (ORs) and 95% confidence intervals (CIs) for factors associated with AMR. The Cox regression model was utilized to estimate the unadjusted hazard ratios (HRs) and 95% CIs of risk factors for mortality within 30 days from bacteremic onset in patients who received antimicrobial treatment at least three days following the start of infection. 

Variables with univariate *p*-values < 0.10 or clinical plausibility were included in the multivariable models to identify independent factors associated with AMR and 30-day mortality. Differences were considered statistically significant at a *p*-value of less than 0.05. All statistical analyses were performed using Stata, version 18.0 (Stata Corp., College Station, TX, USA).

### 4.7. Empirical Antimicrobial Treatment-Decision Algorithm Development

To generate clinically practical algorithms, we placed the significant risk factors for AMR from the multivariate model into several triage steps. The AMR is a serious concern of delayed active empirical treatment. The most optimal algorithm should have high specificity, yet it should lessen the inappropriate empirical broad spectrum antimicrobial treatment. The sensitivity, specificity, positive predictive value (PPV), negative predictive value (NPV), and C-statistic of the algorithm in predicting AMR-BSI were calculated among patients who had bacterial BSI. The algorithm was evaluated in the internal validation cohort from 16 April 2022 to 30 June 2022.

## 5. Conclusions

One-fourth of community-onset BSIs were antimicrobial-resistant. Almost one-third of *Enterobacteriaceae* were non-susceptible to ceftriaxone. Development of a treatment-decision algorithm based on the independent risk factors for AMR-BSI consisting of the presence of MDROs within 90 days, prior antimicrobial use within 90 days, and urinary source could aid in empiric treatment decisions. Reducing excessive broad antimicrobial treatment in NAMR-BSI and increasing useful broad treatment in AMR-BSI would optimize clinical outcomes while reducing the risk of further resistance emergence.

## Figures and Tables

**Table 1:** Baseline characteristics of 450 unique adult patients with AMR-BSI and NAMR-BSI.

Variables	AMR-BSI n = 114 (%)	NAMR-BSI n = 336 (%)	*p* Value
Emergency room	72 (63.2%)	241 (71.7%)	
Outpatient clinic	42 (36.8%)	95 (28.3%)	
Age (years), median (IQR)	74 (57–83)	71 (59–80)	0.56
Male	47 (41.2%)	147 (43.8%)	0.64
**Preexisting medical conditions**			
Chronic pulmonary disease	7 (6.1%)	17 (5.1%)	0.66
Cardiovascular disease	35 (30.7%)	75 (22.3%)	0.07
Chronic liver disease	9 (7.9%)	25 (7.4%)	0.87
Chronic kidney disease	16 (14.0%)	34 (10.1%)	0.25
Neurologic disease	30 (26.3%)	58 (17.3%)	0.04
Diabetes mellitus	41 (36.0%)	127 (37.8%)	0.73
Hypertension	60 (52.6%)	168 (50%)	0.63
Active solid tumor	29 (25.4%)	64 (19.1%)	0.15
Active hematologic malignancies	3 (2.6%)	19 (5.7%)	0.20
HIV	0 (0%)	5 (1.5%)	0.19
Kidney transplantation	8 (7.0%)	11 (3.3%)	0.09
Stem cell transplantation	0 (0%)	2 (0.6%)	0.41
Connective tissue diseases	11 (9.7%)	15 (4.5%)	0.04
Chemotherapy in 6 months	7 (6.1%)	27 (8.0%)	0.51
Corticosteroids at ≥20 mg of prednisone daily or equivalent for ≥14 days	4 (3.5%)	12 (3.6%)	0.98
Post-COVID-19 within 60 days	9 (7.9%)	13 (3.9%)	0.09
Presence of hemodialysis or central venous catheters	9 (7.9%)	35 (10.4%)	0.43
**Severity of acute illness index**			
qSOFA score, median (IQR)	1 (0–2)	1 (0–2)	0.40
Pitt bacteremia score	1 (0–2)	1 (0–2)	0.97
ICU admission following BSIs	21 (18.4%)	80 (23.8%)	0.23
On mechanical ventilator	14 (12.3%)	51 (15.2%)	0.45
On vasopressor	14 (12.3%)	60 (17.9%)	0.17
**Epidemiological risks**			
Prior admission within 90 days	57 (50.0%)	94 (28.0%)	<0.001
Colonization or infection with MDROs during preceding 90 days	40 (35.1%)	27 (8.0%)	<0.001
Ceftriaxone-resistant *Enterobactericeae*	47 (41.2%)	16 (4.8%)	
Carbapenem-resistant *Enterobactericeae*	11 (9.6%)	6 (1.8%)	
Extremely drug-resistant *P. aeruginosa*	6 (5.3%)	3 (0.9%)	
Extremely drug-resistant *A. baumannii*	4 (3.5%)	3 (0.9%)	
**Previous antibiotic exposure within 90 days**	67 (58.8%)	95 (28.3%)	<0.001
Carbapenems	21 (18.4%)	14 (4.2%)	
Ceftriaxone	22 (19.3%)	22 (6.5%)	
Piperacillin-tazobactam	16 (14.0%)	26 (7.7%)	
Fluoroquinolones	15 (13.2%)	16 (4.8%)	
Amoxicillin-clavulanate	10 (8.8%)	17 (5.1%)	
Vancomycin	4 (3.5%)	4 (1.2%)	
Oral third generation cephalosporins	4 (3.5%)	3 (0.9%)	
Ceftazidime	3 (2.6%)	0	
Cefepime	1 (0.9%)	5 (1.5%)	
**Type of identification**			
Unknown primary source	14 (12.3%)	71 (21.1%)	0.04
Pneumonia	4 (3.5%)	16 (4.8%)	0.58
Skin and soft tissue infection	3 (7.5%)	8 (6.0%)	0.72
Bone and joint infection	1 (0.9%)	12 (3.6%)	0.14
Urinary tract infection	58 (50.9%)	100 (29.8%)	<0.001
Hepatobiliary infection	12 (10.5%)	42 (12.5%)	0.58
Intra-abdominal infection	17 (14.9%)	38 (11.3%)	0.31
Infective endocarditis	1 (0.9%)	13 (3.9%)	0.11
Central nervous system infection	0 (0%)	8 (2.4%)	0.10
Catheter-associated bloodstream infection	2 (1.8%)	20 (6.0%)	0.07

Abbreviations: IQR, interquartile range; AMR-BSI, antimicrobial-resistant bloodstream infection; NAMR-BSI, non-antimicrobial-resistant bloodstream infection; MDROs, multidrug-resistant organisms.

**Table 2:** Univariable analysis and multivariable analysis of risk factors for antimicrobial resistance.

Variables	Univariable Analysis		Multivariable Analysis	
	OR (95% CI)	*p*	aOR (95% CI)	*p*
Cardiovascular disease	1.54 (0.96–2.48)	0.07	1.51 (0.88–2.59)	0.13
Neurologic disease	1.71 (1.03–2.83)	0.04	1.40 (0.79–2.46)	0.25
Kidney transplantation	2.23 (0.87–5.69)	0.09	1.65 (0.56–4.80)	0.36
Connective tissue diseases	2.29 (1.02–5.13)	0.05	2.26 (0.91–5.61)	0.08
Prior admission within 90 days	2.57 (1.66–3.99)	<0.001	1.30 (0.73–2.31)	0.37
Presence of MDROs during preceding 90 days	6.19 (3.57–10.72)	<0.001	3.63 (1.95–6.75)	<0.001
Previous antibiotic exposure within 90 days	3.61 (2.32–5.63)	<0.001	1.94 (1.08–3.50)	0.03
Unknown primary source	0.52 (0.28–0.96)	0.04	0.75 (0.37–1.53)	0.43
Urinary source	2.44 (1.58–3.78)	<0.001	1.79 (1.06–3.01)	0.03
CLABSI	0.08 (0.06–1.23)	0.09	0.33 (0.07–1.62)	0.17

Abbreviations: aOR, adjusted odds ratio; CI, confidence interval; MDROs, multidrug-resistant organisms; CLABSI, central line-associated bloodstream infection.

**Table 3:** Hazard ratio for 30-day all-cause mortality for adult patients with community-onset bacterial bloodstream infection.

Variables	Univariable Analysis		Multivariable Analysis	
	HR (95% CI)	*p*	aHR (95% CI)	*p*
Antimicrobial resistance	0.71 (0.31–1.62)	0.41		
Inactive empirical treatment within 24 h	0.67 (0.20–2.19)	0.51		
Pitt bacteremia score	1.42 (1.23–1.63)	<0.001	1.39 (1.20–1.62)	<0.001
Solid malignancy	3.03 (1.53–5.99)	0.001	2.61 (1.30–5.24)	0.01
Hypertension	0.47 (0.23–0.95)	0.04	0.49 (0.24–1.01)	0.054
Urinary source	0.27 (0.10–0.69)	0.006	0.30 (0.11–0.79)	0.02
Pneumonia source	4.86 (1.88–12.57)	0.001	2.05 (0.75–5.56)	0.16

Abbreviations: HR, hazard ratio, CI, confidence interval.

**Table 4:** Appropriateness of antimicrobial treatment of 441 unique adult patients with AMR-BSI vs. NAMR-BSI receiving at least one dose of an antimicrobial agent.

Variables	AMR-BSI	NAMR-BSI	*p* Value
**Empirical antimicrobial treatment**	n = 113 (%)	n = 328 (%)	
Optimally active coverage (appropriate)	79 (69.9)	121 (36.9)	<0.001
Broad spectrum active coverage	0	190 (57.9)	
Inactive spectrum coverage	34 (30.1)	17 (5.2)	
Multiple antimicrobial change in 48 h (range 2–6 times)	27 (23.9)	92 (28.1)	0.39
Unnecessary double coverage	2 (1.8)	21 (6.4)	0.06
**Definitive antimicrobial treatment**	n = 109 (%)	n = 311 (%)	
Optimal drug and duration (appropriate)	36 (33.0)	113 (36.3)	0.71
Narrower/simpler antimicrobial is available	14 (12.8)	57 (18.3)	0.33
Inadequate spectrum coverage	4 (3.7)	2 (0.6)	0.06
Step down to oral treatment is possible	8 (7.3)	42 (12.8)	0.18
Unnecessary double coverage	2 (1.8)	10 (3.2)	0.59
Duration of antimicrobial treatment ^a^, median (IQR)	13 (8–14)	13 (9–14)	0.74
Shorter duration is possible ^a^	52/90 (57.8)	115/229 (50.2)	0.22

Abbreviation: IQR, interquartile range. ^a^ In patients with uncomplicated BSI, and not in palliative care within 5 days.

**Table 5:** Sensitivity, specificity, positive predictive value, negative predictive value, and C-statistics of the algorithm in predicting antimicrobial-resistant infection.

	All BSIs (n = 450)	*Enterobacteriaceae* BSIs (n = 298)	Validation Cohort (n = 227)
Sensitivity (95% CI)	49.1% (36.9–58.7%)	57.5% (46.8–67.6%)	55.2% (41.5–68.3%)
Specificity (95% CI)	85.4% (81.2–89.0%)	81.9% (75.9–86.9%)	93.5% (88.7–96.7%)
PPV (95% CI)	53.3% (45.4–61.1%)	59.3% (50.7–67.2%)	74.4% (58.8–86.5%)
NPV (95% CI)	83.2% (80.4–85.6%)	80.7% (76.6–84.2%)	85.9% (80.0–90.6%)
C-statistic	0.73	0.76	0.77

Abbreviations: CI, confidence interval; positive predictive value, PPV; negative predictive value, NPV; BSIs, bloodstream infections.

## Data Availability

The study dataset is available from the corresponding author upon reasonable request.

## Supplementary Materials

The following supporting information can be downloaded at: https://www.mdpi.com/article/10.3390/antibiotics12121699/s1, Figure S1: Antimicrobial susceptible percentage of clinical isolated bacteria with ≥5 first isolate counts; Table S1: Categories of appropriate and inappropriate antimicrobial use.
