# Supplementary material for: From Epidemiology of Community-Onset Bloodstream Infections to the Development of Empirical Antimicrobial Treatment-Decision Algorithm in a Region with High Burden of Antimicrobial Resistance
Source: Antibiotics (Basel). 2023 Dec 5;12(12):1699. doi: 10.3390/antibiotics12121699 (PMC10740575; doi:10.3390/antibiotics12121699)
Supplement: Supplementary file 1 [file antibiotics-12-01699-s001.zip › antibiotics-2702430-supplementary.pdf]

Supplementary Materials

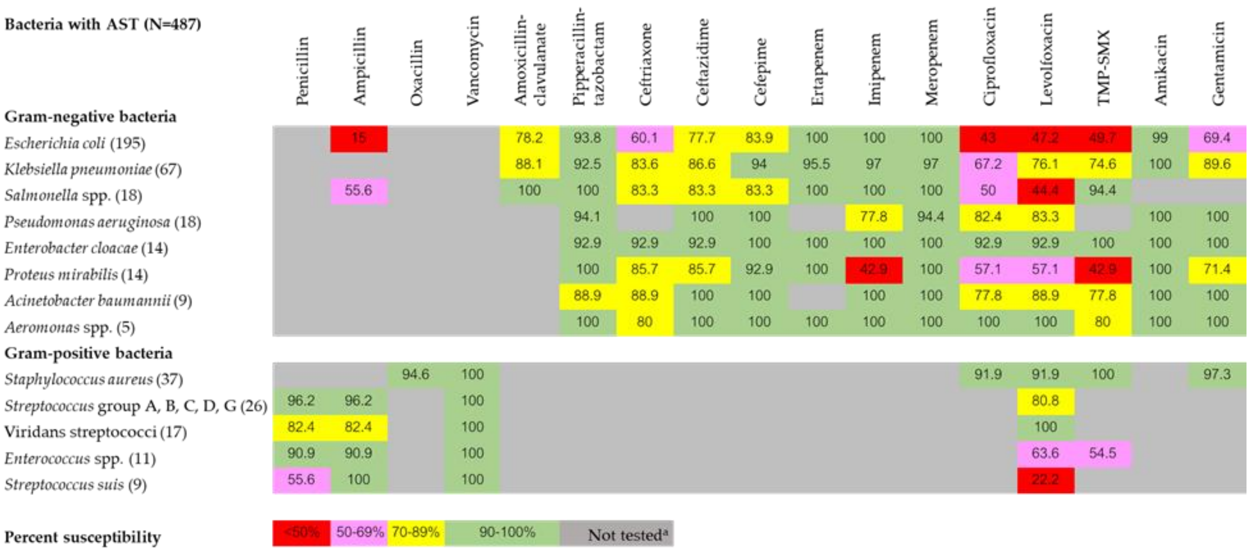

Bacteria with AST (N=487)	Penicillin	Ampicillin	Oxacillin	Vancomycin	Amoxicillin-clavulanate	Pipperacillin-tazobactam	Ceftriaxone	Ceftazidime	Cefepime	Ertapenem	Imipenem	Meropenem	Ciprofloxacin	Levolfoxacin	TMP-SMX	Amikacin	Gentamicin
**Gram-negative bacteria**																	
Escherichia coli (195)		15			78.2	93.8	60.1	77.7	83.9	100	100	100	43	47.2	49.7	99	69.4
Klebsiella pneumoniae (67)					88.1	92.5	83.6	86.6	94	95.5	97	97	67.2	76.1	74.6	100	89.6
Salmonella spp. (18)		55.6			100	100	83.3	83.3	83.3	100	100	100	50	44.4	94.4		
Pseudomonas aeruginosa (18)						94.1		100	100		77.8	94.4	82.4	83.3		100	100
Enterobacter cloacae (14)						92.9	92.9	92.9	100	100	100	100	92.9	92.9	100	100	100
Proteus mirabilis (14)						100	85.7	85.7	92.9	100	42.9	100	57.1	57.1	42.9	100	71.4
Acinetobacter baumannii (9)						88.9	88.9	100	100		100	100	77.8	88.9	77.8	100	100
Aeromonas spp. (5)						100	80	100	100	100	100	100	100	100	80	100	100
**Gram-positive bacteria**																	
Staphylococcus aureus (37)			94.6	100									91.9	91.9	100		97.3
Streptococcus group A, B, C, D, G (26)	96.2	96.2		100										80.8			
Viridans streptococci (17)	82.4	82.4		100										100			
Enterococcus spp. (11)	90.9	90.9		100										63.6	54.5		
Streptococcus suis (9)	55.6	100		100										22.2			

Figure S1. Antimicrobial susceptible percentage of clinical isolated bacteria with ≥5 first isolate counts. Abbreviations: AST, antimicrobial susceptibility testing; TMP-SMX, trimethoprim-sulfamethoxazole.

Table S1. Categories of appropriate and inappropriate antimicrobial use.

Term	Definition
Optimally active coverage (appropriate)	Empirical active antimicrobial treatment based on local antimicrobial guidelines
Broad spectrum active coverage	Empirical active antimicrobial treatment has broader spectrum than in the local antimicrobial guidelines
Inactive spectrum coverage	Empirical inactive antimicrobial treatment according to susceptibility test report
Narrower/simpler antimicrobial is available	Available active narrower spectrum coverage or once daily dosing antimicrobials
Multiple antimicrobials change in 48h	>1 antimicrobial change in 48h, while awaiting for susceptibility test report
Unnecessary double coverage	Combination treatment to cover anaerobes or MRSA in skin and soft tissue infection and biliary tract infection
Shorter duration is possible	Antimicrobials can be stopped in 6-10 days in hemodynamically stable and afebrile patients ≥48 h
Oral step-down treatment is possible	Available active oral antimicrobials in patients with appropriate source control measures, and appropriate clinical response by day 5 of bacteremia

Abbreviations: MRSA, methicillin-resistant *Staphylococcus aureus*.
